# Prevalence of Positive Level IIb Lymph Nodes in Tongue Carcinoma: Experience From a Tertiary Care Center in North India

**DOI:** 10.7759/cureus.23882

**Published:** 2022-04-06

**Authors:** Vishnu Saigal, Ravi Meher, Praveen K Rathore, Raman Sharma, Nita Khurana

**Affiliations:** 1 ENT and Head & Neck Surgery, Maulana Azad Medical College and Lok Nayak Hospital, New Delhi, IND; 2 Pathology, Maulana Azad Medical College and Lok Nayak Hospital, New Delhi, IND

**Keywords:** spinal accessory nerve, neck dissection, metastasis, lymph nodes, carcinoma tongue

## Abstract

Introduction

Complications during and after dissection of level IIb lymph nodes include spinal accessory nerve (SAN) dysfunction, which results in the limitation of shoulder movements and, thus, hurts the quality of life. The current study aims to know the occurrence of level IIb lymph node positivity in tongue carcinoma.

Methods

This cross-sectional study was conducted from January 2019 to December 2019 in a tertiary care center in North India. Adult cases with primary ulcer-proliferative growth over the lateral border of the tongue were included in the study. The level IIb lymph node positivity from the postoperative histopathology report was the primary outcome measure of this study. To investigate the potential association of tumor size on level IIb lymph node positivity, we compared the maximum tumor dimensions among the level IIb lymph node-positive and -negative groups. In addition, to analyze the impact of the tumor's invasive nature on level IIb lymph node positivity, we compared the depth of invasion and proportion of cases with muscle involvement among the level IIb lymph node-positive and -negative groups. Lastly, to investigate their concurrent occurrences, we compared the number of level IIb lymph node-positive cases among the level IIa lymph node-positive and -negative groups.

Results

A total of 39 patients fulfilling the inclusion criteria were included in the study. Only six had positive level-IIb lymph nodes. No significant associations of tumor size, invasion depth, muscle invasion, or involvement of level IIa lymph nodes with the positivity of level IIb lymph nodes were found. However, only three were level IIb lymph node-positive in 28 level IIa lymph node-negative cases.

Conclusion

Considering the low risk of isolated level IIb lymph node positivity in level IIa lymph node-negative cases, the dissection of level IIb nodes could be omitted during the surgical excision of the tumor. However, radiological investigations detecting metabolic activity should be used in the preoperative period and postoperative follow-up to detect early lymph node involvement and disease recurrence.

## Introduction

The appropriate management of the neck in patients with tongue cancer remains an area that has not been well defined. There is a lack of consensus regarding the type of management in patients with a node-negative neck, while it is well known that a node-positive neck needs a modified radical neck dissection (MRND) [1]. Level IIb lymph nodes contained in the sub-muscular recess are the lymph nodes lying over the fascia of the splenius capitis and levator scapulae, related superiorly to the spinal accessory nerve (SAN), anteromedially to the sternocleidomastoid muscle, and inferiorly to the skull-base [2]. Complications that may arise during and after level IIb node dissection include SAN dysfunction, which limits shoulder movements and, thus, affects the quality of life. To overcome this complication, avoiding the dissection of level IIb nodes has been proposed [3]. Understanding the prevalence of level IIb lymph node positivity can potentially influence the surgical management and prognosis of tongue carcinomas. The current study aims to analyze the occurrence of level IIb lymph node positivity and nodal metastasis in patients affected by tongue carcinoma. We also analyzed the impact of the tumor's invasive nature and level IIa lymph node positivity on the positive status of level IIb lymph nodes.

## Materials and methods

This cross-sectional study was conducted from January 2019 to December 2019 at the otorhinolaryngology department of Maulana Azad Medical College and associated Lok Nayak Hospital, New Delhi, India. All patients attending the department's outpatient clinics and with ulcer-proliferative growth of the lateral border of the tongue were included in the study. We included only adult patients, who were primary cases of squamous cell carcinoma (SCC) of the tongue with or without clinically palpable neck nodes. Patients having a large tumor crossing midline or involving the mandible or base of the tongue and patients with a recurrence of malignancy and carcinoma of the tongue after chemo-radiotherapy were excluded from the study. A detailed history and examination were carried out. A biopsy of the primary tumor mass was performed in all cases to establish the diagnosis. Contrast-enhanced computed tomography (CECT) of the base of the skull to the diaphragm was obtained to know the extent of the disease and to rule out any distant metastasis. After clearance from the anesthesia department and informed written consent, surgical excision of the primary tumor with appropriate neck dissection (supra-omohyoid, extended supra-omohyoid, or modified radical neck dissection) was carried out. Level IIb lymph node was excised in all the cases. The level IIb lymph node positivity from the postoperative histopathology report was the primary outcome measure of this study. Besides this, we noted the demographic characteristics of the patients, such as age, gender distribution, laterality, risk factors, tumor staging, lymph nodes positivity of other levels, distribution of positive lymph node groups among the included patients, and the type of carcinoma based upon histopathological features.

To investigate the potential association of tumor size with level IIb lymph node positivity, we compared the maximum tumor dimensions among the level IIb lymph node-positive and -negative groups. To analyze the impact of the tumor's invasive nature on level IIb lymph node positivity, we compared the depth of invasion and proportion of cases with muscle involvement among the level IIb lymph node-positive and -negative groups. Lastly, to investigate their concurrent occurrences, we compared the number of level IIb lymph node-positive cases in the level IIa lymph node-positive and -negative groups. The continuous variables were presented as mean±standard deviation (SD), the discrete variables were presented as numbers, and the categorical variables were presented as percentages. For comparing continuous variables, we used an unpaired t-test. For comparing proportions, we used the Chi-square test. A p-value of less than 0.05 was considered statistically significant.

## Results

A total of 39 patients fulfilling the inclusion criteria were included in the study. Their detailed demographic characteristics are provided in Table 1. The clinical and operative findings are presented in Table 2. Finally, the comparative data between the cases with level IIb positive and negative lymph nodes has been presented in Table 3.

**Table 1:** Demographic characteristics of the tongue carcinoma cases reviewed in the current study.

Characteristics	Observations
Age (years)	41.97±12.35
Number of male patients, n (%)	33 (84.6%)
Number of cases with right-sided involvement ratio	25 (64.1%)
Percentage distribution of risk factors:	
Tobacco chewing	66.6%
Bidi smoking	20.5%
Cigarette smoking	5.1%
Alcohol consumption	5.1%

**Table 2:** The clinical and operative findings of the tongue carcinoma cases reviewed in the current study.

Clinical/ operative findings	Observations
Tumor staging (out of 39 cases):	
Number of patients with stage T1, n (%)	14 (35.9%)
Number of patients with stage T2, n (%)	18 (46.1%)
Number of patients with stage T3, n (%)	1 (2.6%)
Number of patients with stage T4a, n (%)	6 (15.4%)
Lymph nodes staging (out of 39 cases):	
Number of patients with stage N1, n (%)	20 (51.2%)
Number of patients with stage N2a, n (%)	15 (38.5%)
Number of patients with stage N2b, n (%)	4 (10.3%)
Levels of positive lymph nodes:	
Positive level Ia positive lymph nodes (out of 95 nodes biopsied)	4 (4.2%)
Positive level Ib positive lymph nodes (out of 134 nodes biopsied)	10 (7.46%)
Positive level IIa positive lymph nodes (out of 202 nodes biopsied)	20 (9.90%)
Positive level IIb positive lymph nodes (out of 180 nodes biopsied)	16 (8.89%)
Number of patients with positive lymph nodes:	
Level Ia positive lymph node cases, n (%)	4 (10.2%)
Level Ib positive lymph node cases, n (%)	5 (12.8 %)
Level IIa positive lymph node cases, n (%)	8 (20.5%)
Level IIb positive lymph node cases, n (%)	6 (15.4%)

**Table 3:** The comparison of tumor invasiveness among the level IIb lymph node-positive and -negative groups.

Variable	Level IIb lymph node-positive cases (n = 6)	Level IIb lymph node-negative cases (n = 33)	Statistical significance
Mean maximum tumor dimension (in cm)	1.93±0.67 cm	2.43±1.17 cm	p = 0.36 (statistical insignificant)
Mean depth of invasion (in cm)	0.70±0.20 cm	0.81±0.40 cm	p = 0.82 (statistical insignificant)
Number of cases with muscle invasion, n (%)	4 (66.7%)	26 (78.7%)	p = 0.51 (statistical insignificant)
Number of concurrent level IIa lymph node-positive cases (n = 11)	3	8	p = 0.19 (statistical insignificant)
Number of concurrent level IIa lymph node-negative cases (n = 28)	3	25	

All patients were diagnosed histopathologically with SCC. It was well-differentiated in 18 (46.2%) patients, while in the remaining 21 (53.8%) subjects, it was moderately differentiated. The patients with lymph node positivity rates in moderately and well-differentiated SCC were classified into different groups. There were no patients diagnosed with poorly differentiated SCC.

## Discussion

The current study was conducted to investigate the occurrence of level IIb lymph node positivity in tongue carcinoma subjects. As the tongue has a very rich and peculiar lymphatic drainage, carcinoma primarily metastasizes through lymphatic drainage. Nodal metastasis of tongue cancer is quite predictable, which majorly depends on the site and size of the lesion and clinical staging of cancer. Level I, II, and III lymph nodes are the most affected. Chances of skip metastasis and occult metastasis are less than 5%, which occurs after the involvement of the oral cavity [4]

In the present study, we had 39 tongue SCC cases. These cases had to undergo appropriate neck dissection. During surgeries, different levels of neck lymph nodes were explored and sent for biopsy. Our analysis suggested that 21 patients were negative for nodal metastasis. Metastases to lymph nodes were found in only 18 patients. More than 50% of negative lymph nodes depict either early access to clinical care by the patient or timely intervention. Li et al. conducted a retrospective data analysis of 161 oral SCC cases with negative cervical lymph nodes [5]. Out of the 161 cases, 87 were tongue cancer cases in their study. Among those 87 cases, only 16 cases presented with lymph node metastasis. In the present study, we had excised 937 lymph nodes from 39 patients with tongue cancers. Most excised lymph nodes were from levels IIa and IIb. On average, 24 different levels of lymph nodes were cleared from each patient. A total of 67 (7.15%) lymph nodes were found positive in 18 cases with an average of 3.7 lymph nodes per patient. Skip metastases were not found in any of the patients.

The available literature suggests that the involvement of IIb lymph nodes in oral cancer averages up to 6%. Lim et al. reviewed 74 patients with a clinically lymph node-negative neck who underwent supraomohyoid neck dissection for SCC of the oral cavity and found a prevalence of metastases at level IIb in 5% of cases [6]. Elsheikh et al. found an incidence of 10% at level IIb lymph nodes [7]. In our study, level IIb lymph node involvement was around 15%, higher than that in the available literature. If we had avoided the dissection of the IIb lymph nodes, then there were chances of recurrence in almost 15% of the cases. Postoperative complications like shoulder dysfunction may be managed as these are not life-threatening, whereas cancer recurrence may become life-threatening for the patient. Elsheikh et al. reported the incidence of occult metastasis to level IIb in tongue cancer patients to be 22% [7]. They concluded that level IIb should be dissected in cases of tongue cancer.

Similarly, de Vicente et al. [3], Pantvaidya et al. [8], and Maher et al. [9] reported the incidence of IIb lymph node to be around 5-6% in tongue cancer cases. Our study findings are different from the findings of Lim et al. [6] as they reported zero isolated IIb lymph nodes. But in our findings, we had two cases with isolated IIb lymph node-positive cases. However, we could not conclude whether the chances of recurrences will be there if we preserve the area. Dabholkar et al. [10] conducted a prospective observational study in which 10.4% of cases were found with positive IIb lymph nodes. The study reported nil isolated or contralateral metastasis of IIb lymph nodes, concluding that IIb lymph nodes may be preserved in clinically lymph node-negative cases. Still, therapeutic neck dissection should be conducted in case of level IIa lymph node-positive. Although in our study, we were found isolated metastasis to IIb, such incidence was very low (10.7%). The addition of non-invasive measures like positron emission tomography-computed tomography (PET-CT) can help know the activity status of these lymph nodes in the preoperative period and during follow-up if they have been preserved.

The current study has the limitations of small sample size and lack of follow-up due to its cross-sectional nature. In addition, the clinical outcomes were not analyzed as the methodology investigated mainly the level II lymph node status based on the histopathological picture. Although there was no significant association that IIb lymph nodes were more commonly positive in positive IIa lymph nodes compared to negative IIa lymph nodes, considering the low positivity rate, the dissection of level IIb nodes could be omitted if the level IIa lymph nodes are negative as it will provide a significant decrease in operative time and less of SAN trauma-related complications. On the other hand, if the level IIa lymph node is positive, it is important to dissect level IIb. However, when omitting the level IIb lymph nodes, close follow-up of the patients through non-invasive radiological investigations like PET-CT is warranted to predict recurrence and lymph node involvement.

## Conclusions

The prevalence of positive level IIb lymph nodes in tongue carcinoma is low. Additionally, the level IIb lymph node positive status may not indicate the tumor size and its invasive nature. Considering the low risk of isolated level IIb lymph node positivity in level IIa lymph node negative cases, the dissection of level IIb nodes can be omitted during the surgical excision of the tumor. However, radiological investigations detecting metabolic activity should be used in the preoperative period and postoperative follow-up to detect early lymph node involvement and disease recurrence. This practice can potentially decrease the operative time and reduce the risk of SAN injury-related complications. Further, large sample studies in different populations will help strengthen these findings.
